# Religiousness Measured by the Four Basic Dimensions of Religiousness Scale (4-BDRS) among Polish Believers: Measurement Quality, Personality and Well-being Correlates

**DOI:** 10.1007/s10943-025-02450-z

**Published:** 2025-09-23

**Authors:** Piotr Szydłowski, Ewa Topolewska-Siedzik, Jan Cieciuch

**Affiliations:** Institute of Psychology, Cardinal Stefan Wyszyński University in Warsaw, Ul. Wóycickiego 1/3 Bud. 14, 01-938 Warsaw, Poland

**Keywords:** 4-BDRS, Religiousness, Personality, Well-being

## Abstract

The Four Basic Dimensions of Religiousness Scale (4-BDRS) was developed within the framework of cross-cultural psychology to measure four universal dimensions of religiousness: believing, bonding, belonging, and behaving. This paper presents the Polish version of the 4-BDRS and reports two studies that examined its measurement quality and the personality and well-being correlates of these dimensions. The scale has demonstrated satisfactory reliability and factorial validity. All dimensions showed theoretically consistent associations with the other measures of religiousness. No significant gender differences (or only weak effects) were found across the four dimensions, whereas the scale scores significantly increased with age. All dimensions were positively related to the personality traits associated with socialization (stability). Moreover, all the dimensions showed a positive association with well-being. Belonging and bonding were more strongly related to well-being than believing and behaving. Specifically, belonging was the most crucial dimension for general and social well-being, whereas bonding was the most important dimension for emotional and psychological well-being. The theoretical implications of these findings are discussed.

## Introduction

The Four Basic Dimensions of Religiousness Scale (4-BDRS), developed by Saroglou (2011), operationalizes the essential dimensions of religiousness. The scale has been adapted to 15 languages across various cultures (e.g., Aditya et al., 2021; Dimitrova, 2014; Dimitrova & Domínguez Espinosa, 2017a; Kumar et al., 2021; Saroglou et al., 2020). We present two studies on the Polish adaptation of the 4-BDRS and expand, on the one hand, knowledge on religiousness in Poland and, on the other hand, knowledge on the personality and well-being correlates of the dimensions differentiated in the 4-BDR model. Poland is a country where 84.5% of the population currently identifies with the Catholic Church and where religion continues to play a prominent role in the lives of its inhabitants (Statistics Poland, 2021), despite ongoing secularization processes (Mariański, 2021; Mariański et al., 2023), especially among young individuals (Pew Research Center, 2018). For the first time, we present the factorial structure of the dimensions measured by the 4-BDRS, their gender and age differences, their relationships to religiousness dimensions differentiated in other models, and their personality and well-being correlates among Polish Christians.

### The 4-BDR Model and Measurement

Saroglou (2011) characterized religiousness as a global orientation toward what people do in connection with what they view as (external) transcendence. To describe religiousness from a cross-cultural perspective, (Sarogluou 2011; Saroglou et al., 2020) differentiated four basic dimensions that are psychologically informed, do not use the theological language of specific religious traditions, can be used to study both universals and specifics across religions and cultures, and have distinct validity, implying partly distinct psychological processes, predictors, and outcomes. These dimensions are believing, bonding, behaving, and belonging. They correspond to the cognitive, emotional, moral, and sociopsychological aspects of religiousness (motives, processes, functions, and results).

*Believing* refers to what people consider to be (external) transcendence. However, in a broad view, it includes religious meaning-making processes and affinities between being religious and holding other beliefs, such as basic world assumptions, just-world beliefs, and beliefs in the meaningfulness of the world, as well as of one’s personal life. *Bonding* is an emotional dimension that includes self-transcendent experiences that bind a person to what that person perceives as transcendent reality, particularly during a ritualized framework such as private prayers, meditations, or public ceremonies in churches, mosques, synagogues, or other “sacred” places. *Behaving* is concerned with morality, specific norms, and moral arguments that defy the right and wrong from a religious perspective. In general, this aspect refers to actions based on instructions, such as those found in God’s commandments and the sacred texts. *Belonging* describes what it means to belong to a religious community or group. In this community, individuals satisfy their need to belong to, maintain, and benefit from a social identity (Saroglou, 2011).

The quality of the model and measurement have been verified across different countries (Aditya et al., 2021; Dimitrova, 2014; Dimitrova & Domínguez Espinosa, 2017a; Kumar et al., 2021; Saroglou et al., 2020). However, studies on the 4-BDRS in Eastern Europe, a region with its own distinct historical and cultural characteristics, are lacking, and our research aims to fill this gap.

### Religiousness Measured by 4-BDR and other Characteristics of Religiousness

To answer how the four basic dimensions measured by the 4-BDRS are related to other models, we focus on two established models: the Commitment–Reflectivity Circumplex (CRC) model developed by Krauss & Hood Jr. (2013) and the Centrality of Religious Model (CRM) proposed by Huber (2003) based on Glock and Stark (1965).

The CRC model posits that religious orientation measures differ primarily in terms of the level of commitment and reflectivity they capture. In other words, these measures vary in the extent to which they assess dedication to religious faith (committed vs. uncommitted) and the extent to which belief systems are subject to analysis, questioning, and growth (reflective vs. unreflective) (Krauss & Hood Jr, 2013). Consequently, orientations can be categorized into four quadrants: committed/reflective, committed/unreflective, uncommitted/reflective, and uncommitted/unreflective, with some being further divided into more nuanced orientations. All 10 orientations differentiated by the CRC are presented in Appendix A.

The CRM proposed by Huber (2003) and based on Glock and Stark (1965) distinguishes five dimensions: interest in religious issues, indicating cognitive involvement in the elaboration of religious content; religious beliefs, indicating the degree of certainty about the existence of a transcendental reality; prayer, illustrating the frequency of contact with a transcendental reality and the subjective importance of personal contact with transcendence; religious experience, which determines how often transcendence is present in a person’s everyday life; and worship, indicating the frequency and subjective importance of participation in religious services. In our research, for the first time, we related the dimensions measured by the 4-BDRS to the CRC and CRM models.

### Religiousness Measured by 4-BDR and Personality

To understand the personality underpinnings of the four basic dimensions, Saroglou et al. (2020) used the Big Five model and showed that agreeableness and conscientiousness were positively correlated with all four dimensions of religiousness, and that the belonging dimension was positively associated with extraversion and negatively related to openness to experience.

The Big Five model has been criticized in the literature, and in order to solve the problems identified with the Big Five, (Cieciuch & Strus 2017; Strus et al., 2014) proposed the Circumplex of Personality Metatraits (CPM). The CPM uses two broad personality dimensions (Alpha/Stability and Beta/Plasticity) called metatraits or higher-order personality factors, already discovered and described by Digman (1997) & DeYoung et al. (2002). The CPM applies the idea of circular organization, arranging Alpha/Stability and Beta/Plasticity as orthogonal axes within a circumplex structure. In addition, the CPM incorporates two other metatraits, Gamma/Integration and Delta/Restraint, which are located orthogonally to each other and at a 45-degree rotation to the Alpha/Stability and Beta/Plasticity. All metatraits differentiated by CMC are presented in Appendix B. In our research, we describe, for the first time, the personality underpinnings of religiousness measured by the 4-BDRS using CPM.

### Religiousness Measured by 4-BDR and Well-being

A few studies have provided initial evidence regarding the links between religiousness, measured by the 4-BDRS, and overall well-being. At the correlational level, all four dimensions are positively related to well-being (Aditya et al., 2021; Dimitrova & Domínguez Espinosa, 2017a, 2017b). However, regressions controlling for the common variance between the four show that bonding and belonging have unique and additive links in predicting higher well-being, whereas believing has a unique opposite role in predicting decreased well-being (Saroglou et al., 2020).

In our research, we used the model proposed by (Karaś et al., 2014; Keyes & Waterman, 2003) to describe the relationship between religiousness measured by the 4-BDRS and well-being. He differentiated three facets of well-being: emotional well-being (associated with the experience of positive feelings and emotions, such as happiness, interest in life, and life satisfaction), psychological well-being (resistance to external pressures, a sense of agency and competence, an individual’s pursuit of personal growth, the experience of warm and trusting interpersonal relationships, holding beliefs and convictions that provide life with purpose and meaning, and the acceptance of one’s strengths and weaknesses), and social well-being (beliefs in being a valuable member of society, social acceptance, concern for understanding the world—including perceptions of the quality, organization, and functioning of the social reality—and the conviction that society is recognizable, meaningful, and predictable).

## Current Studies

In two studies, we developed a Polish version of the 4-BDRS and provided information on its psychometric quality (an earlier Polish version of the scale was used as part of an international study, without validation information being provided; Saroglou et al., 2020). Moreover, we present gender and age differences in the dimensions of religiousness, differentiation in their importance, their relations to two other models of religiousness (CRC and CRM), personality underpinnings (using the CMP), and well-being correlates among Polish believers.

We formulated the following hypotheses:

1. Consistent with other studies using the 4-BDRS (e.g., Aditya et al., 2021; Dimitrova, 2014; Saroglou et al., 2020), we expected to confirm the high reliability of the scales and the validity of the four-factor solution.
2. We expect that in the Polish group, a different pattern of mean importance across the 4-BDRS dimensions will emerge compared to Catholic groups from other countries, where believing was found to have the highest significance (Saroglou et al., 2020). Polish religiousness is unique compared to that of other countries in the region. Until 1989, Poland was under a communist and atheistic system, and the Catholic Church was one of the main opposition centers focused on rituals, a kind of social life, and organizing people around them. This suggests that religiousness is still rooted in ritual and social practices and engagement in a religious community, with less emphasis on experiencing personal emotions related to transcendence. Therefore, we anticipate a pattern in which the means of believing, belonging, and behaving are significantly higher than that of bonding.
  Regarding gender and age differences, we formulated the following hypothesis:
3. Although some studies report a higher level of women's religiousness among Christians (Dimitrova & Domínguez Espinosa, 2017a) or Hindus (Kumar et al., 2021), the majority of studies report no gender differences among Christians (Aditya et al., 2021; Kumar et al., 2021; Saroglou et al., 2020). Therefore, we expect no gender differences in the 4-BDRS dimensions among Polish believers, who are predominantly Christian.
4. So far, no studies have examined age group differences using the 4-BDRS; however, numerous studies have shown that younger generations are significantly less inclined than older generations to identify with a religion, believe in God, or participate in diverse religious traditions (Kasselstrand et al., 2023; Pew Research Center, 2018). According to the Pew Research Center (2018), Poland, like other economically developing countries, is undergoing secularization processes, where an “age gap in religious commitment” can be observed, meaning that people up to approximately 40 years of age are less committed to and less engaged in institutional religion than are older people. We expect that this "age gap" will also be observed in the Polish group for religiousness as measured by the 4-BDRS.

Regarding the relationship with other models of religiousness, we formulated the following hypotheses:

- We anticipate that the four dimensions of religiousness measured by the 4-BDRS as predictors of religious orientation will reveal a distinct configuration of the four quadrants of the CRC model.
- Believing is characterized by both certainty in religious beliefs and the pursuit of understanding religious doubts. Therefore, we hypothesized that this dimension would positively explain religious orientations from three quadrants: committed/unreflective, committed/reflective, and uncommitted/reflective, and negatively from the uncommitted/unreflective quadrant.
- Bonding, due to its positive emotional nature arising from participation in religious rituals and community activities, is expected to be a positive predictor of religious orientations in the committed/unreflective and committed/reflective quadrants, where individuals remain attached to religious truths while actively developing their faith. Since bonding is closely tied to religious community involvement, we hypothesized that it would negatively predict the uncommitted/unreflective and uncommitted/reflective quadrants, which reflect uncertainty about the objectivity of religious beliefs.
- Behaving emphasizes adherence to religious moral norms within a religious community and is therefore expected to be a predictor of the committed/unreflective quadrant and a negative predictor of the uncommitted/reflective quadrant. Within this dimension, there is no doubt or uncertainty regarding the objective validity of the religious norms. Due to its strong positive association with the need for cognitive closure (Saroglou et al., 2020), this dimension is characterized by a clear understanding of religious doctrines that may not necessarily be internalized as personal beliefs. This, in turn, encourages conformity with the social environment, making it a predictor of the uncommitted/unreflective quadrant.
- Belonging, which emphasizes the role of religious communities in personal religiousness, is expected to be a positive predictor of religious orientation from the committed/unreflective quadrant and a negative predictor of religious orientation from the uncommitted/reflective quadrant.
- We expect positive relationships between all dimensions of religiousness measured by the 4-BDRS and distinguished in the CRM proposed by Huber (2003).

Regarding the relationship between personality and well-being, we formulated the following hypotheses:

(H7) Considering the theoretical background of CPM and the results on the relations between dimensions of religiousness measured by 4-BDRS and the Big Five personality traits, we assumed positive relationships of all basic dimensions of religiousness with three metatraits located above the Beta dimension (Delta-Plus/Self-Restraint, Alpha-Plus/Stability, and Gamma-Plus/Integration), and negative relationships with metatraits located below the Beta dimension (Delta-Minus/Sensation-Seeking, Alpha-Minus/Disinhibition, and Gamma-Minus/Disharmony). The Alpha dimension is formed by the shared variance of agreeableness, conscientiousness, and emotional stability. Previous studies have linked all dimensions of religiousness to conscientiousness and agreeableness (Saroglou, 2010; Saroglou et al., 2020). However, some dimensions of religiousness are positively related to extraversion and negatively related to openness to change (Saroglou, 2010; Saroglou et al., 2020). Because the Beta dimension is formed by the shared variance of these two traits, the relations between religiousness dimensions can cancel each other out in relation to Beta. Moreover, considering the circular arrangement of metatraits in the CPM, we expected a sinusoidal pattern of relationships between all dimensions of religiousness and metatraits, with the highest positive correlations with Alpha-Plus and the highest negative correlations with Alpha-Minus.
(H8) We hypothesized that bonding and belonging mainly explain well-being. This expectation is justified by the sharing of positive emotions in relation to transcendence, particularly through participation in communal rituals in the case of bonding, and by the direct support provided by religious community life in the case of belonging.

Two studies were conducted to verify the hypotheses. To ensure robust and replicable results, we verified H1 (factorial validity and reliability) and H2 (mean importance) separately in both studies: H3 (gender differences) and H4 (age differences) on a joint sample from both studies. We verified H5 (related to CRC) and H7 (related to personality) in Study 1, and H6 (related to CRM) and H8 (related to well-being) in Study 2.

Koenig and Carey (2024, 2025) cautioned against analyzing variables measured by contaminated scales, particularly in relation to health-related constructs. In our study, we analyzed the relationships between religiousness, measured using the 4-BDRS, and other variables describing religiousness, personality, and well-being. Contamination was not an issue in this study because the dimensions of religiousness assessed by the 4-BDRS are defined and measured in a completely different way than personality and well-being. For the other measures of religiousness used, there is some similarity to the 4-BDRS, but this is due to the fact that these are different instruments and models addressing similar conceptualizations of religiousness. Therefore, their convergence was expected and interpreted as evidence of convergent validity.

## Method

### Sample and Procedure

In both studies, the data were gathered online using the snowball method via social media, that is, Facebook. The studies aimed to evaluate the religiousness of only those who self-identified as believers in God. In the first study, 544 adults participated (*M*_*age*_ = 42.31, *SD* = 15.26, female: 57%), and the vast majority of participants were Catholic (99.8%). Educational attainment was generally high: 36.0% held a master’s degree, 22.2% had completed postgraduate education beyond the master’s level, and 10.8% reported a bachelor’s degree. Additionally, 29.4% had completed secondary education, 1.3% had completed only primary education, and 0.2% had no formal education. Regarding employment status, 63.6% of the participants were employed full-time, 2.9% were employed part-time or worked casually, 11.4% were students, 2.9% were on a leave of absence, 5.5% were unemployed, and 13.6% were either retired or receiving a disability pension. Relationship status varied across the sample: 50.6% were married, 9.4% were in informal (cohabiting) relationships, 28.9% were single and not in a relationship, and 3.1% were widowed. In addition, 5.1% were identified as diocesan priests and 2.9% as members of religious orders (monks or nuns). In terms of place of residence, 62.3% of the participants lived in cities with more than 100,000 inhabitants, 10.5% in cities with 50,000–100,000 residents, 13.2% in towns with fewer than 50,000 residents, and 14% in rural areas.

A total of 537 adults participated in the second study. However, as the study aimed to evaluate the religiousness of only those who self-identified as God believers, data from six participants who identified as atheists, agnostics, or deists were excluded from the study. The final sample size was 531 adults (*M*_*age*_ = 36.87, *SD* = 14.05, female: 48.40%), of whom the vast majority were Catholic (99.1%), followed by Protestants (0.6%), Orthodox Christians (0.2%), and spiritual but not religious (0.2%). Educational attainment was relatively high: 33.3% of the participants reported holding a master’s degree, 16.4% had completed postgraduate education beyond the master’s level, and 15.3% held a bachelor’s degree. Additionally, 31.5% reported having completed secondary education, and 3.6% had completed only primary education. None of the participants reported formal education. In terms of employment status, most respondents (62.9%) were employed full-time. Part-time or casual workers accounted for 3.8% of the sample, while 17.1% were students. A smaller portion of the sample was on a leave of absence (2.8%), unemployed (6.4%), retired, or receiving disability pensions (7.0%). Regarding marital and relationship status, 41.4% of the participants were married, and 11.3% were in informal cohabiting relationships. A total of 38.8% reported being single with no partner, and 1.9% were widowed. In addition, 4.0% of the respondents identified themselves as diocesan priests and 2.6% as members of religious orders (monks or nuns). Regarding place of residence, 59.3% of the sample reported living in large cities (with more than 100,000 inhabitants). The remaining participants lived in towns with fewer than 50,000 residents (16.2%), medium-sized cities (12.2%), or rural areas or villages (12.2%).

## Measures

### The Four Basic Dimensions of Religiousness Scale

The 4-BDRS (Saroglou, 2011; Saroglou et al., 2020) was used in both studies. The scale is a 12-item measure of four dimensions of religiousness: believing, bonding, behaving, and belonging, with three items per dimension. Participants rated their agreement with each statement on a seven-point Likert scale ranging from 1 = *totally disagree* to 7 = *totally agree*.

In preparing the Polish version of the 4-BDRS, we followed the criteria for psychometric validation and translation of religious and spiritual measures formulated by Koenig and Al Zaben (2021). Permission was obtained from the author of the 4-BDRS. Two authors of this paper prepared independent forward translations and discussed them to reach a reconciled version. Next, an independent back-translation was conducted. Two authors also conducted a cognitive debriefing, and the back-translation was approved by the author of the 4-BDRS. Following the recommendations of Koenig and Al Zaben (2021), in this study, we present the factorial structure (confirmatory factor analysis), reliability (Cronbach’s alpha), and validity (relations to other models of religiousness, age and gender differences, and relations to personality and well-being) of the 4-BDRS. Appendix C presents the original English items and their Polish translations.

### The Circumplex Religious Orientation Inventory

The Circumplex Religious Orientation Inventory (CROI; Krauss & Hood Jr, 2013) was used in Study 1. The CROI comprises 10 subscales, as listed in Table 1. The inventory contains 63 items scored on a five-point Likert scale ranging from 1 = *strongly disagree* to 5 = strongly agree. The Cronbach’s alpha reliabilities of the scales in the current study ranged between .63 and .89 (see Appendix D).

**Table 1:** Model-fit indices from confirmatory factor analysis of 4-BDRS

	*χ*2 (df)	CFI	RMSEA [90% CI]	SRMR
*Study 1*				
One-factor model	432.940 (54)	.812	.114 [.104; .124]	.064
Four-factor model	143.116 (48)	.953	.060 [.049; .072]	.043
Higher-order factor model	152.273 (50)	.949	.061 [.050; .073]	.045
*Study 2*				
One-factor model	379.447 (54)	.774	.107 [.097; .117]	.074
Four-factor model	83.809 (48)	.975	.037 [.024; .051]	.034
Higher-order factor model	89.439 (50)	.973	.039 [.025; .051]	.037

$\chi^{2}$ Chi-square, *df* degrees of freedom, *CFI* comparative fit index, *RMSEA* root-mean-square error of approximation, *SRMR* standardized root-mean-square residual

### The Centrality of Religiosity Scale

The Centrality of Religiosity Scale (CRS-15, Huber, 2003) with a Polish adaptation by Zarzycka (2007) was used in Study 2. The scale allows the measurement of five dimensions differentiated in CRM by Huber (2003): interest, religious beliefs, prayer, religious experience, and worship. It is also possible to use an overall index of religiosity centrality, which is the mean of all five scales. Participants answered on a five-point Likert scale, but the meaning of particular scale points differed between the questions. For items 1 to 7, the scale ranged from 1=*not at all* to 5=*very*; for items 8 to 13, it ranged from 1=*never* to 5=*very frequently*; for item 14, a seven-point Likert scale (from 1 = *never* to 7 = *a few times per week*); and for item 15, a nine-point Likert scale (from 1 = *never* to 9 = *several times per day*). The Cronbach’s alpha in the current study ranged from .70 to .89 (see Appendix D).

### The Circumplex of Personality Metatraits Questionnaire

Study 1 used the Circumplex of Personality Metatraits Questionnaire—Short Form (CPM-Q-SF (Strus & Cieciuch, 2017) was used in Study 1. This allows the measurement of the eight metatraits listed in Table 2. It consists of 72 items rated on a five-point Likert scale ranging from 1 = *completely disagree* to 5 = *completely agree*. The Cronbach’s alpha reliability of the eight CPM-Q-SF scales in the current study ranged from .64 to .84 (see Appendix D).

**Table 2:** The results from linear regression analysis where religiousness dimensions predict religious orientations

	*t*	*β*	*p*		*t*	*β*	*p*
Personal				Centrality			
*R*^2^ = .26; *F* = 63.40, *p* < .001				*R*^2^ = .48, *F* = 127.98, *p* < .001			
Believing	.19	3.91	< .001	Believing	.35	8.83	< .001
Bonding				Bonding	.13	3.05	.002
Behaving	.18	3.24	.001	Behaving	.09	1.97	.049
Belonging	.23	4.21	< .001	Belonging	.25	5.33	< .001
Gain				Social			
*R*^2^ = .10, *F* = 30.45, *p* < .001				Nonsignificant model			
Believing				Believing			
Bonding				Bonding			
Behaving	.15	2.55	.011	Behaving			
Belonging	.20	3.46	< .001	Belonging			
Doubt				Tentativeness			
*R*^2^ = .09, *F* = 18.85, *p* < .001				*R*^2^ = .19, *F* = 64.16, *p* < .001			
Believing	.20	3.84	< .001	Believing			
Bonding				Bonding	− .23	− 4.54	< .001
Behaving	− .26	− 4.25	< .001	Behaving			
Belonging	− .16	− 2.73	.007	Belonging	− .26	− 5.07	< .001
Dialog				Interest			
*R*^2^ = .08, *F* = 15.96, *p* < .001				*R*^2^ = .19, *F* = 64.48, *p* < .001			
Believing	.17	3.17	.002	Believing	.23	5.14	< .001
Bonding	− .16	− 3.10	.002	Bonding	.28	6.30	< .001
Behaving	− .23	− 4.07	< .001	Behaving			
Belonging				Belonging			
Punishment				Obligation			
*R*^2^ = .01, *F* = 6.21, *p* = .013				*R*^2^ = .06, *F* = 13.04, *p* < .001			
Believing				Believing	− .19	− 3.54	< .001
Bonding				Bonding	− .22	− 4.21	< .001
Behaving	.11	2.49	.013	Behaving	.20	3.52	< .001
Belonging				Belonging			

*R*^2^ are corrected. Empty cells mean particular 4-BDRS scales are not significant predictors and were eliminated from the analysis.

### The Mental Health Continuum-Short Form

Well-being in Study 2 was assessed using the Mental Health Continuum-Short Form (MHC-SF), adapted into Polish by Karaś et al. (2014). It allows for the measurement of well-being and its emotional, psychological, and social facets. It consists of 14 items. Participants indicated the frequency with which they experienced various symptoms of well-being on a six-point Likert scale ranging from 1 = *never* to 6 = *every day*. The Cronbach’s alpha in this study ranged from .82 to .88 (see Appendix D).

## Results

### Reliability (Study 1 and Study 2)

As a preliminary analysis, we computed the descriptive statistics (Appendix E) and intercorrelations of the 4-BDRS (Appendix F). Moderate positive correlations were observed between all four 4-BDRS dimensions. Cronbach’s alphas ranged from .76 to .83 in Study 1 and from .70 to .81 in Study 2. In conclusion, all four scales from the 4-BDRS were characterized by acceptable reliability (understood as internal consistency), as all reached the .70 threshold.

### Factorial Validity (Study 1 and Study 2)

To verify the hypothesis of the four-factor structure of the 4-BDRS, we conducted a confirmatory factor analysis (CFA). Considering the factor analyses of the 4-BDRS presented in the literature (i.e., Aditya et al., 2021; Dimitrova, 2014; Kumar et al., 2021), we tested three CFA models using the 12 items as observed variables: (1) the general factor of religiousness, where one latent variable is loaded by all 12 items; (2) four-factor models in which latent variables are loaded by the respective items in line with the 4-BDRS; and (3) a higher-order model where the four factors from the second model load on a general higher factor of religiousness. We assessed the fit of the models using the most widely applied indices: the root-mean-square error of approximation (RMSEA; recommended < .08), comparative fit index (CFI; recommended < .95), and standard mean square residual (SRMR; recommended < .08) (Hu & Bentler, 1999). The fit indices for all the models are presented in Table 1.

The results suggest that the one-factor model has a visibly worse fit than the other two models, which obtained comparable values for the model-fit indices. Furthermore, adding a higher-order factor of general religiousness did not increase the fit of the model with four factors. The parsimony rule should be considered, which states that if adding an additional factor does not lead to a significant increase in the model fit, a simpler model should be chosen. This suggests that among the compared models, the best model is the one that discriminates the four religiousness scales, as originally proposed (Saroglou, 2011; Saroglou et al., 2020). It is worth noting that the results of Study 1 and Study 2 are very similar, which supports the robustness of the conclusion.

The results of the CFA for the four-factor model with correlations between each latent variable and the factor loadings of items used in the 4-BDRS are presented in Appendix G. All presented correlations are significant with *p* < .001.

### Differences in Mean Importance of the 4-BDRS (Study 1 and Study 2)

To examine the expectation of mean differences between the BDRS-4 dimensions, we conducted a repeated-measures ANOVA, following the approach of Saroglou et al. (2020). Mauchly’s test of sphericity was significant; therefore, the Greenhouse–Geisser correction was applied to adjust for violations of the sphericity assumption in estimating differences between the 4-BDRS scale levels. The ANOVA results were significant (Study 1: *F*(2.80, 1521.18) = 20.79, *p* < .001, *η*^2^ = .11; Study 2: F(2.70, 1431.48) = 24.51, *p* < .001, *η*^2^ = .04). Post hoc comparisons with Sidak’s correction for multiple comparisons revealed that the bonding dimension (Study 1: *M* = 5.32; Study 2: *M* = 3.98) was significantly lower than all other 4-BDRS scales, namely believing (Study 1: *M* = 5.56; Study 2: *M* = 4.19), behaving (Study 1: *M* = 5.68; Study 2: *M* = 4.24), and belonging (Study 1: *M* = 5.62; Study 2: *M* = 4.18), with no statistically significant differences between the latter three dimensions. As expected, the results suggest that a lower level of bonding than other religiousness dimensions is characteristic of the Polish sample. It is worth noting that the results of Study 1 and Study 2 are very similar, which supports the robustness of the conclusion.

### Gender and Age Differences (Study 1 and Study 2)

To check whether there were gender differences in the level of the 4-BDRS scales, a series of *t* tests was performed on joint samples from Study 1 and Study 2. The results are presented in Appendix H. It turned out that there is only a weak difference in belonging between women and men; thus, we can conclude that in the Polish sample, there are no meaningful gender differences in religiousness dimensions.

To verify age differences in the levels of the 4-BDRS scales, a one-way ANOVA was conducted. Age was recorded on a five-point scale, where 1 = 18–30 years, 2 = 31–40 years, 3 = 41–50 years, 4 = 51–60 years, and 5 = 61 years or older. Appendix I presents the gender and age group distributions.

**Fig. 1:**
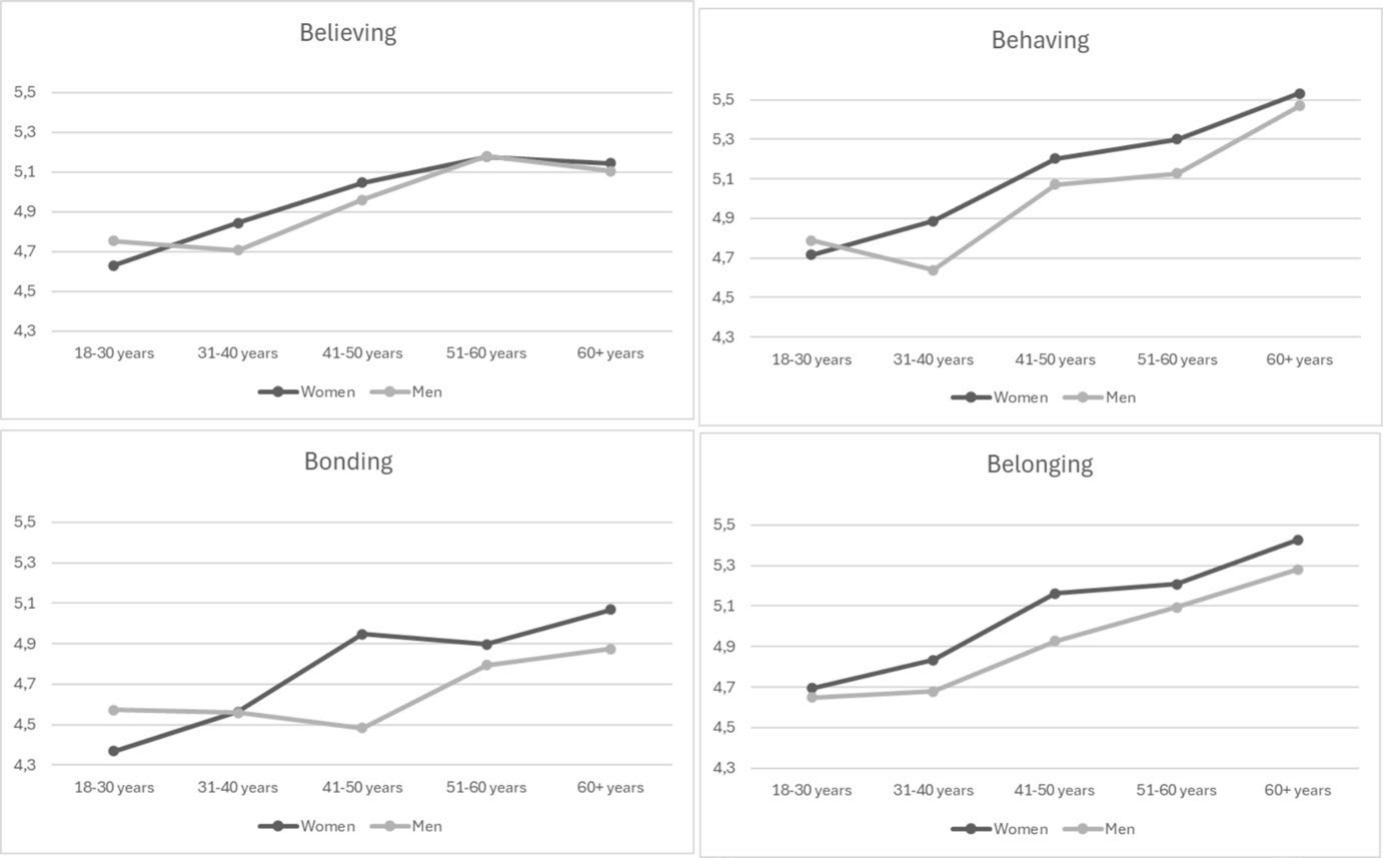
Means of religiousness dimensions measured by 4-BDRS in age groups

The result of Levene’s test of variance homogeneity was significant (*p* < .05) for all four variables, indicating a lack of homogeneous variances. Thus, in the analysis, Welsh’s amendment was used to assess the effect of age group on four religiousness dimensions. The results show that the level of all variables, namely believing *F*_*Welsh*_(4,397.99)=5.78, *p*<.001, *η*^2^=.021, bonding *F*_*Welsh*_(4,401.99)=5.99, *p*<.001, *η*^2^=.021, behaving *F*_*Welsh*_(4,398.14) = 12,87, *p* < .001, *η*^2^=.049, and belonging *F*_*Welsh*_(4,400.39)=9.98, *p*<.001, *η*^2^=.037, varied between age groups. Considering the assumed increase in the level of religiousness with age, we used the Helmert contrast to check how age groups differed. This method compares the mean of a particular factor level (except for the last one) with the mean of the subsequent levels. Helmert’s contrast showed in the case of believing significant comparisons for age groups 18–30 years (*p* < .001) and 31–40 years (*p* < .001), which indicates that younger participants present lower levels of belonging, and from the age group 41–50 years to the oldest ones, the difference is not significant. The contrast comparisons for bonding suggest similar age differences where the 18–30 age group (*p* < .001) and 31–40 years (*p* = .001) age groups were significantly different from the older groups. Helmert’s contrast revealed that belonging followed the same pattern of differences, where the 18–30 age group (*p* < .001) and 31–40 years (*p* = .001) significantly varied from older age groups. Behaving levels differed even to a greater degree, where besides the 18–30 age group (*p* < .001) and 31–40 years (*p* = .001) also 41–50 age group (*p* = .038) present lower levels than older participants. The means of the 4-BDRS scales, separately in gender and age groups, are presented in Fig. 1.

These results show that all religiousness dimensions differed between age groups, with an increase in some older groups compared with younger ones.

### Dimensions of Religiousness and Religious Orientations (Study 1)

To verify our expectations regarding the relationships between the dimensions of religiousness measured by the 4-BDRS and the religious orientations distinguished in the CRC (Krauss & Hood Jr, 2013), we first conducted a correlation analysis of the 4-BDRS and CROI scales, followed by a regression analysis. The correlation coefficients are presented in Appendix J.

All dimensions of religiousness were weakly to moderately positively associated with centrality, personal, interest, and gain, and negatively associated with tentativeness. Bonding, behaving, and belonging are negatively related to dialog and doubt. Believing and bonding were negatively associated with obligation, whereas behaving and belonging were positively associated with punishment.

To check how believing, bonding, behaving, and belonging enable particular religious orientations to be predicted, we conducted a multivariate linear regression analysis using a backward elimination data introduction method. The criterion for predictor elimination was *p* > .05. The backward elimination method starts with a model containing all potential independent variables and then gradually removes those variables that contribute the least to the model fit. Thus, it enabled us to obtain a model with only significant predictors when the predictor elimination threshold was > .05. For each CROI variable, a separate analysis was conducted in which the four dimensions of the 4-BDRS were predictors. To enhance clarity, only models containing the final set of predictors are reported. Table 2 presents these results.

Believing was the strongest positive predictor of centrality, doubt, and interest, followed by personal and dialog, and a negative predictor of obligation. Similarly, bonding was the strongest positive predictor of interest, followed by centrality, and the strongest negative predictor of obligation, followed by tentativeness and dialog. Behaving was the strongest positive predictor of punishment and obligation, followed by gain, personal, and centrality, while it was the strongest negative predictor of doubt and dialog. Belonging was the strongest positive predictor of personal gain, followed by centrality, and the strongest negative predictor of tentativeness, followed by doubt. The results enable us to positively verify hypothesis H5a regarding the positive predictive power of believing in religious orientations from three quadrants: committed/unreflective, committed/reflective, and uncommitted/reflective, and negative for religious orientation from uncommitted/unreflective quadrants. Similarly, H5b on positive predictions from bonding to religious orientations in the committed/unreflective and committed/reflective quadrants and negative predictions to uncommitted/unreflective and uncommitted/reflective quadrants were confirmed. Based on the analysis, we confirmed that behaving positively predicts the committed/unreflective and uncommitted/unreflective quadrants and negatively predicts the uncommitted/reflective quadrant, which confirms hypothesis H5c. As stated in H5d, we confirmed that belonging is a positive predictor of religious orientation in the committed/unreflective quadrant and a negative predictor of religious orientation in the uncommitted/reflective quadrant.

### Relationship with Centrality of Religiousness (Study 2)

The relationships between religiousness dimensions measured by the 4-BDRS and the variables from the CRM were analyzed using Pearson’s correlations. The results are presented in Appendix K. All dimensions measured by 4-BDRS were positively and moderately linked to all variables distinguished in CRM, which enables us to confirm H6.

### Dimensions of Religiousness and Personality Metatraits (Study 1)

**Fig. 2:**
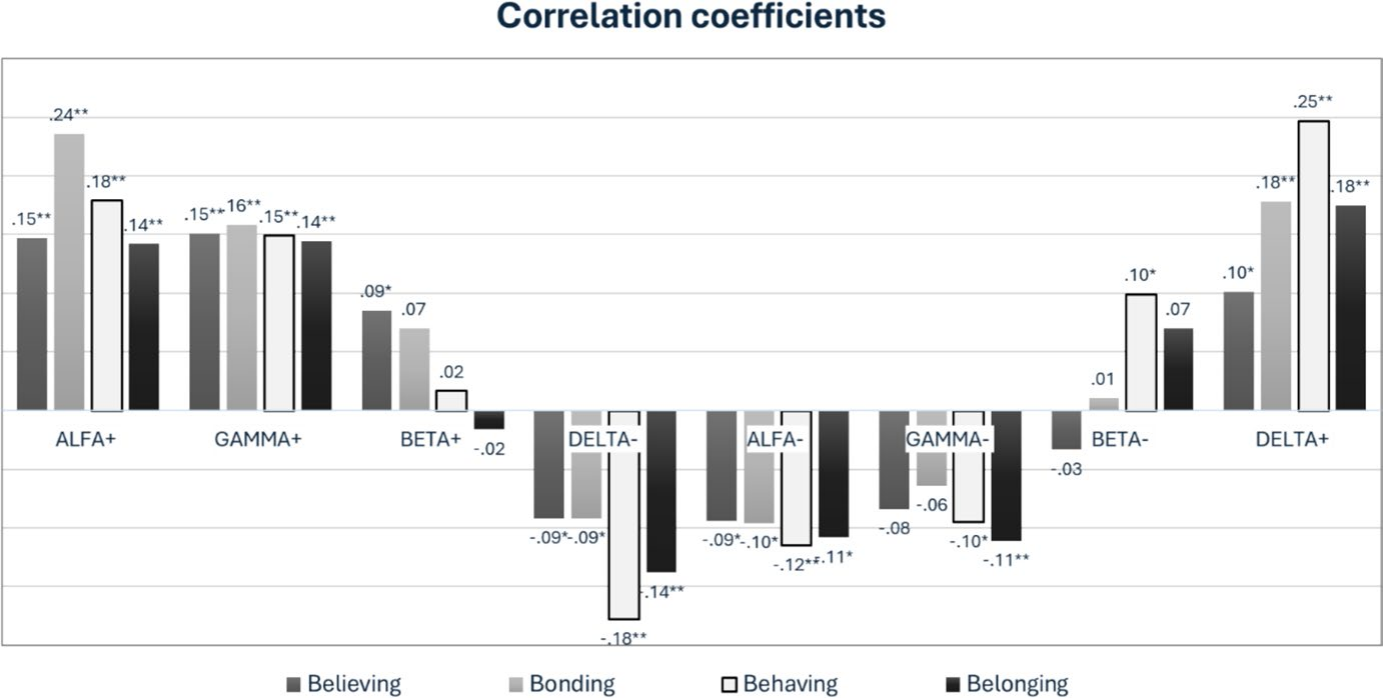
Correlations between religiousness dimensions and personality metatraits

The relationships between religiousness dimensions measured by the 4-BDRS and personality traits from the CPM were analyzed using Pearson’s correlations. The results are presented in Fig. 2.

As expected, the observed relationships between religiousness dimensions and personality metatraits followed a sinusoidal pattern. Specifically, correlation coefficients are more similar for metatraits adjacent to the circumplex and differ more substantially as the distance between metatraits increases; moving further along the circumplex, at some point, the correlations become close to zero and then negative. As expected, metatraits below the beta dimension were negative, and those above the beta dimension were positive. It was revealed that the correlations with both pools of Beta were indeed nonsignificant, with two exceptions of very small but statistically significant correlations (believing with Beta-Plus and behaving with Beta-Minus). The highest correlations of believing and bonding were expected with Alpha-Plus, while the correlations of behaving and belonging were the second highest with Alpha-Plus and the highest with Delta-Plus (a metatrait located as a neighbor to the Alpha-Plus on the circumplex). These results are generally in line with expectations, with a small shift in the highest correlations of the two dimensions from Alpha to Delta.

### Dimensions of Religiousness and Well-being (Study 2)

Appendix L presents the correlations between the religiousness dimensions and well-being. Bonding, behaving, and belonging were weakly and positively correlated with all scales of well-being (only believing was not correlated with well-being). To check the importance of the four commonly considered dimensions of religiousness for well-being in a more detailed way, a multivariate linear regression analysis with a backward elimination data introduction method was conducted on the data. A separate analysis was conducted for each aspect of well-being, in which the four religious variables were predictors. The criterion for predictor elimination was *p* > 0.5. The results are presented for the last model, which contains only the significant predictors. The results are presented in Table 3.

**Table 3:** Results from the multivariate linear regression models with well-being facets as a dependent variable and religiousness dimensions as predictors

General			
	*t*	β	*p*
*Adjusted R*^*2*^ = .023*; F* = 13.25*, p* < .001			
Belonging	3.64	.16	< .001
Social			
*Adjusted R*^*2*^ = .040*; F* = 23.28*, p* < .001			
Belonging	4.83	.21	< .001
Emotional			
*Adjusted R*^*2*^ = .009*; F* = 5.76*, p* = .017			
Bonding	2.40	.10	.017
Psychological			
*Adjusted R*^*2*^ = .010*; F* = 6.23*, p* = .013			
Bonding	2.50	.11	.013

The results suggest that the 4-BDRS variables are rather weak predictors of well-being, but the relationship pattern varies between the well-being aspects. First, religiousness variables were the strongest predictors of social well-being, and only to some extent (only for models with a reduced number of predictors) did the 4-BDRS play a role in emotional and psychological well-being. When 4-BDRS interrelationships are considered, which is the case for a regression analysis, it is possible to indicate which aspect of religiousness is especially important for a particular aspect of well-being. Thus, for general and social well-being, the most important (and among others, the only significant) aspect of the 4-BDRS was belonging, while for emotional and psychological well-being, it was bonding. The results confirm that the belonging and bonding dimensions are the most important predictors of well-being, as expected in H8.

## Discussion

We present, for the first time, the measurement quality of the 4-BDRS in Poland, as well as the relationship between the dimensions measured by the 4-BDRS and other variables describing religiousness, personality underpinnings, and well-being correlates. We found that the 4-BDRS is a reliable and valid instrument for measuring the basic dimensions of religiousness. In Poland, where Catholicism remains the predominant religion, a four-factor structure was replicated. Our results further support the model distinguishing believing, bonding, behaving, and belonging from a cross-cultural perspective as a model that describes religiousness well in the context of Christianity and other religions (e.g., Aditya et al., 2021; Dimitrova, 2014; Dimitrova & Domínguez Espinosa, 2017a; Kumar et al., 2021; Saroglou, 2011; Saroglou et al., 2020).

We examined differences in the mean importance of the 4-BDRS and found differences from the results reported by Saroglou et al. (2020). According to Saroglou et al. (2020), in contexts where Catholicism plays a dominant role, the typical hierarchy is believing > bonding, behaving, and belonging. According to our results, in Poland, the hierarchy is believing, behaving, and belonging > bonding. Notably, none of the religious cultural zones identified by Saroglou et al. (2020) exhibited this pattern. Such a relatively low level of bonding indicates Polish specificity, with a lesser role for emotional experiences in contact with transcendence.

No significant gender differences were observed. This aligns with prior research on religiousness in Poland (e.g., Zarzycka et al., 2024) and Christian communities in cultural contexts distinct from Eastern Europe utilizing the 4-BDRS (e.g., Aditya et al., 2021). However, the 4-BDRS significantly differentiated the age groups. Our study confirmed the existence of an “age gap in religious commitment,” with younger generations being less engaged in religious practices and institutional religious affiliation than older generations. This suggests that secularization processes are present in Poland and are aligned with global trends (Pew Research Center, 2018). In countries experiencing economic progress, differentiation, rationalization, religiousness, and religious authority tend to decline over time (Kasselstrand et al., 2023).

In summary, in relation to our samples, which are highly religiously engaged and regularly participate in religious services, this may reflect the persistence of a more traditional model of religious functioning—one centered around rituals rather than deeply experienced spirituality, which is regarded as a more personal aspect of faith. We consider this related to the specificity of Polish religiousness. For nearly 50 years, the communist system, which was oppressive toward the Catholic religion and believers in Poland, shaped religious engagement in Poland. Attachment to the Church, religious practices, and an emphasis on the community were the primary ways of relating to transcendence. The focus on ensuring the survival of religion as such, through fidelity to religious practice, hindered the development of other forms of religiousness, particularly those that would be less dependent on ecclesiastical authority or orthodox religious doctrine and more personal and internalized. However, this model is subject to changes driven by ongoing secularization processes. Our study confirmed the existence of an age gap in the Polish context, where individuals over the age of approximately 40 years are more attached to institutionalized religion than younger generations.

Furthermore, the obtained results confirmed the theoretically expected relationships between CROI and the four dimensions of religiousness measured by the 4-BDR scale. Previous research (Saroglou et al., 2020) has demonstrated that believing exhibits noteworthy plasticity and a paradox: “This dimension seemed to underline fundamentalism, but it was also the only one that uniquely predicted high existential quest” (p. 569). In our study, we confirmed the existence of this paradox: Believing positively predicts religious orientations from as many as three quadrants of the CRC model, covering ¾ of the circular model. Our findings highlight the core of this paradox in the following ways. The sphere of beliefs and meaning-making processes underlies a broad range of religious orientations; however, specific religious orientations differentiate and shape the particular nature of an individual’s religious beliefs. Thus, religious beliefs associated with religious orientations in the uncommitted/reflective quadrant pertain to religious doubts, particularly during times of personal difficulty. Meanwhile, beliefs corresponding to religious orientations in the committed/reflective quadrant are linked to in-depth reflection on religious topics, whereas those in the uncommitted/unreflective quadrant are characterized by firm adherence to closure beliefs and reluctance to change them. Believing was also found to negatively predict religious orientation in the uncommitted/unreflective quadrant, where a person primarily experiences social pressure to appear or behave in a religious manner.

Bonding positively predicted two religious orientations from two quadrants of the CRC model: committed/reflective and committed/unreflective, and negatively predicted by the other two quadrants: uncommitted/unreflective and uncommitted/reflective. Thus, it serves as a positive predictor of religious orientations in which commitment and fidelity to tradition are central (committed), while differentiating them based on the degree to which belief systems are analyzable and open to questioning and growth (reflective versus unreflective). At the same time, bonding was also a negative predictor of religious orientations, in which commitment to tradition was not considered important, and the extent of critical reflection on religious beliefs ranged from unreflective to reflective.

Behaving positively explains religious orientations from two quadrants of the CRC model: committed/unreflective and uncommitted/unreflective, and negatively predicts orientations from the uncommitted/reflective quadrant. Therefore, it serves as a positive predictor for religious orientations in which there is no openness to interpreting religious norms (unreflective), but which differ in the degree of engagement in religious practice and fidelity to tradition (committed vs. uncommitted). Additionally, behaving was a negative predictor of religious orientation situated in the quadrant where individuals value the search for new meaning and seek a deeper understanding of religious messages. Based on previous research, we know that this dimension is a predictor of a high need for closure, high authoritarianism, low existential questioning, and openness to experience (Saroglou et al., 2020, 2022). Consequently, behaving seems to be an indicator of the rigid aspect of religiousness, in which there is no room to search for solutions with a wide scope of reference, apart from those that are “imposed” in religion as part of, for example, the commandments.

Belonging positively explains religious orientations from the committed/unreflective quadrant of the CRC model and negatively from the uncommitted/reflective quadrant. Thus, it serves as a predictor of religious orientations that emphasize adherence to religious commandments and tradition (committed/unreflective) while simultaneously rejecting critical reflection on them (uncommitted/reflective).

All 4-BDRS dimensions were positively and moderately associated with all facets of the centrality of religion in an individual’s life.

Regarding the personality underpinnings of the dimensions of religiousness, we confirmed our expectations regarding the relationships between these dimensions and the metatraits distinguished in the CPM. All dimensions were positively and weakly associated with the metatraits Delta-Plus, Alpha-Plus, and Gamma-Plus and negatively associated with Delta-Minus, Alpha-Minus, and Gamma-Minus, displaying the expected sinusoidal pattern of correlations. The results showed that bonding correlated positively and most strongly with Alpha-Plus, which reflects a general tendency toward socialization manifested in social adaptation, an ethical stance toward the world, and the ability to delay gratification, self-motivation, and perseverance. Behaving was most strongly and positively associated with Delta-Plus, which is linked to high behavioral control and conventionalism. Behaving also showed a weak positive association with Beta-Minus, which is characterized by submissiveness in interpersonal relationships, cognitive and behavioral passivity, and a certain degree of inhibition and stagnation. Believing was the only dimension that correlated positively and weakly with Beta-Plus, which encompasses cognitive and behavioral openness to change, engagement in new experiences, a tendency toward exploration, initiative and inventiveness in social relationships, and a focus on personal development.

Finally, we demonstrated the relationship between the 4-BDRS and well-being. We observed correlations between bonding, behaving, and belonging with well-being, consistent with previous studies (Aditya et al., 2021; Dimitrova & Domínguez Espinosa, 2017a, 2017b). However, we did not find any association with believing. After conducting a regression analysis, we found that only the bonding and belonging dimensions were predictors of well-being, consistent with the findings of Saroglou et al. (2020). The dimension of belonging is connected to general and social well-being, whereas that of bonding is linked to psychological and emotional well-being. The findings validate that the belonging dimension encompasses an aspect of religiousness in which the religious community plays a prominent role. Bonding refers to the enjoyment, meaning, and fulfillment that individuals experience when they engage in personal rituals, such as private prayers, meditations, or public ceremonies in religious settings, such as churches, mosques, synagogues, or other sacred sites. In light of the existing literature (Saroglou et al., 2020; Tokarz & Łowicki, 2024), we know that collective forms of religiousness can enhance an individual’s well-being through the presence of nonreligious factors such as social support, belongingness, and identification. The religious community plays a significant role in both bonding and belonging among Christian groups. Based on our study, we can theorize that bonding individuals derive satisfaction from religious rituals, which creates a need for the presence of a community (emotional, psychological well-being, and interest orientation). In belonging, the community is needed primarily for personal, nonreligious benefits, such as a sense of being recognized (social well-being and gain orientation).

## Limitations

This study had some limitations that must be considered in future studies. Although these two groups are diverse in terms of age, they are dominated by people under 40 years old. In the future, it would be worth extending the research to include groups of people who are not sufficiently covered in this study. What characterizes a group up to the age of 40, including their religiousness, may change over time. Since this sample is characterized by a very high commitment to religious practices, it would be beneficial in the future to include people with moderate commitment, as well as those who consider themselves spiritual but not religious. Longitudinal research is required to test the possible impact of religiousness on well-being.

## Conclusions

This paper presents the Polish version of the 4-BDRS and two studies on measurement quality as well as personality and well-being correlates of these dimensions. The tool’s reliability and factorial validity were sound. All dimensions are theoretically related to other variables that describe religiousness. No meaningful gender differences were found; however, a significant increase in scale levels with age was observed. All dimensions were positively related to personality metatraits, displaying a sinusoidal pattern of relations, with the highest relations to Alpha-Plus and Delta-Plus. Moreover, the relationships between the four dimensions and well-being were all positive. The belonging and bonding dimensions had a stronger effect on well-being than the believing and behaving dimensions. Finally, the study indicated that for general and social well-being, the most crucial aspect of the 4-BDRS is belonging; whereas for emotional and psychological well-being, it is bonding.

## Data Availability

The data that support the findings of this study are available in OSF at 10.17605/OSF.IO/UY276.

## Appendix A

Definitions of the variables measured by the circumplex religious orientation inventory

**Table:**

Quadrant	Religious orientation	Definition
Committed/unreflective	Punishment	An approach to religion that is heavily colored by a fear of God, by conscious attempts to avoid divine punishment, and a belief that negative events are controllable as well as meaningful
	Gain	The degree to which religion is approached as a method for gaining wealth, health, success, and other personal desires
	Centrality	The degree to which religion is important and central to an individual’s life
	Personal	An approach toward religion in order to gain comfort, protection, forgiveness, and help in general
Committed/reflective	Interest	The amount to which an individual enjoys learning, reading, and talking about religion and religious concepts
Uncommitted/reflective	Dialog	The degree to which an individual is aware that their religion is affected by “the contradictions and tragedies of life”
	Tentativeness	The degree to which an individual is self-critical and uncertain of the objective validity of their beliefs about religion (whether religious or nonreligious)
	Doubt	The degree to which an individual enjoys and values their religious doubts, uncertainties, and questions
Uncommitted/unreflective	Social	The degree to which a person involves themselves in religion in an effort to make or see friends and others as social acquaintances
	Obligation	The amount to which a person feels social pressure to act or be religious

The table is based on Krauss and Hood (2013).

## Appendix B

Definitions of metatraits from the circumplex of personality metatraits

**Table:**

Metatraits	Definition
Gamma-Plus (integration)	Well-being, a warm and prosocial attitude toward people, both intra- and interpersonal harmony, openness to the world in all its richness, and effectiveness in attaining important goals
Beta-Plus (plasticity)	Cognitive and behavioral openness to change and engagement to new experiences, a tendency to explore, initiative and invention in social relations, as well as orientation toward personal growth
Delta-Minus (sensation-seeking)	Broadly defined impulsiveness, high emotional lability, stimulation-seeking, provocativeness, and expansiveness in interpersonal relations
Alpha-Minus (disinhibition)	High level of anti-social tendencies underpinned by unrestraint and low frustration tolerance, as well as aggression and antagonism toward people, social norms, and obligations
Gamma-Minus (disharmony)	Inaccessibility (distrust, coldness, distance) in interpersonal relationships, depressiveness, pessimism, and proneness to suffer from psychological problems
Beta-Minus (passiveness)	Apathy, submissiveness in interpersonal relations, cognitive and behavioral passivity, as well as some type of inhibition and stagnation
Delta-Plus (self-restraint)	Low emotionality (both negative and positive), high behavior control, a tendency to adjust oneself, conformism, and conventionality
Alpha-Plus (stability)	Stability in the emotional, motivational, and social functioning, expressed as a general social adaptation tendency, an ethical attitude toward the world, and the ability to delay gratification, motivate oneself, and perseverance

Table after Strus and Cieciuch (2017).

## Appendix C

English and polish version of 4-BDRS

4-Basic dimensions of religiousness scale (4-BDRS).

**Table:**

You may be interested or not in religion for a variety of reasons. Please try to be as specific as possible in your answers to the following questions dealing with the reasons they eventually make you to be interested or not on religion					
			Totally disagree (1)	(2)…(6)	Totally agree (7)
1	Believing	I feel attached to religion because it helps me to have a purpose in my life			
2		It is important to believe in a Transcendence that provides meaning to human existence			
3		Religious beliefs have important implications for our understanding of human existence			
4	Bonding	I like religious ceremonies			
5		Religious rituals, activities, or practices make me feel positive emotion			
6		Religion has many artistic, expressions, and symbols that I enjoy			
7	Behaving	I am attached to the religion for the values and ethics it endorses			
8		Religion helps me to try to live in a moral way			
9		When I've got a moral dilemma, religion helps me to make a decision			
10	Belonging	In religion, I enjoy belonging to a group/community			
11		Belonging to a religious tradition and identifying with it is important for me			
12		Referring to a religious tradition is important for my cultural/ethnic identity			

The table is adapted from Saroglou et al., (2020).

4-Basic dimensions of religiousness scale (4-BDRS).

**Table:**

Z różnych powodów możesz być zainteresowany lub niezainteresowany religią. Poniżej znajdują się stwierdzenia dotyczące takich powodów. Proszę ustosunkować się do każdego z nich tak dokładnie, jak to możliwe					
			Całkowicie się nie zgadzam (1)	(2)…(6)	Całkowicie się zgadzam (7)
1	Believing	Czuję się związany z religią, ponieważ pomaga mi to odnaleźć cel w życiu			
2		To ważne, aby wierzyć w Transcendencję, która nadaje sens ludzkiej egzystencji			
3		Wierzenia religijne mają istotne znaczenie dla zrozumienia ludzkiej egzystencji			
4	Bonding	Lubię ceremonie religijne			
5		Rytuały, aktywność i praktyki religijne wywołują we mnie wiele pozytywnych emocji			
6		Religia wyraża się w wielu artystycznych formach i symbolach, które lubię			
7	Behaving	Jestem związany z religią ze względu na wartości i etyke, które religia głosi			
8		Religia pomaga mi w staraniach, aby żyć moralnie			
9		Kiedy przeżywam dylematy moralne, religia pomaga mi podjąć decyzję			
10	Belonging	Cieszę się, że przynależę do grupy/wspólnoty religijnej			
11		Przynależność do tradycji religijnej i identyfikacja z nią są dla mnie ważne			
12		Odniesienie do tradycji religijnej jest ważne dla mojej tożsamości kulturowej/etnicznej			

## Appendix D

Cronbach’s alpha coefficients of other scales used in Study 1 and Study 2

**Table:**

Variable	Cronbach’s alpha
The circumplex of personality metatraits questionnaire	
Alfa-Plus	.64
Gamma-Plus	.80
Beta-Plus	.77
Delta-Minus	.76
Alfa-Minus	.77
Gamma-Minus	.84
Beta-Minus	.71
Delta-Plus	.66
The circumplex religious orientation inventory	
Personal	.63
Centrality	.89
Gain	.66
Social	.77
Doubt	.73
Tentativeness	.74
Dialog	.88
Interest	.86
Punishment	.79
Obligation	.73
The centrality of religiosity scale	
Total	.89
Interest	.81
Beliefs	.85
Prayer	.77
Experience	.86
Worship	.70
The mental health continuum-short form	
Total	.92
Emotional	.86
Psychological	.88
Social	.82

## Appendix E

Descriptive statistics and Cronbach’s alpha coefficients of 4-BDRS scales

**Table:**

	Study 1			Study 2		
	*M*	*SD*	α	*M*	*SD*	*α*
1. Believing	5.56	1.30	.76	4.19	.81	.70
2. Bonding	5.32	1.26	.83	3.98	.70	.75
3. Behaving	5.68	1.17	.80	4.24	.72	.81
4. Belonging	5.62	1.32	.79	4.18	.72	.71

## Appendix F

Intercorrelations of 4-BDRS scales in Study 1 and Study 2

**Table:**

	Study 1				Study 2			
	1	2	3	4	1	2	3	4
1. Believing	–				–			
2. Bonding	.49*	–			.37*	–		
3. Behaving	.59*	.60*	–		.44*	.50*	–	
4. Belonging	.56*	.64*	.70*	–	.35*	.54*	.60*	–

**p* < .001.

## Appendix G

Confirmatory factor analysis of the 4-BDRS four-dimensional structure in Study 1

Confirmatory factor analysis of the 4-BDRS four-dimensional structure in Study 2

## Appendix H

Gender differences in religiousness dimensions (df = 1073)

**Table:**

Variable	Women		Men		*t*
	*M*	*SD*	*M*	*SD*	
Believing	4.92	1.31	4.84	1.26	1.03
Bonding	4.72	1.25	4.59	1.20	1.65
Behaving	5.06	1.21	4.87	1.21	2.52
Belonging	5.01	1.31	4.79	1.25	2.76*

**p* < .01.

## Appendix I

Gender and age groups distribution in joint sample from Study 1 and Study 2

**Table:**

	Age groups						General
		18–30 ys	31–40 ys	41–50 ys	51–60 ys	above 60 ys	
Gender	Woman	158	114	129	91	74	566
	Man	196	138	93	50	32	509
General		354	252	222	141	106	1075

## Appendix J

Correlation coefficients between religiousness dimensions and religious orientations

**Table:**

	Believing	Bonding	Behaving	Belonging
Punishment	.03	.02	.11*	.09*
Gain	.21**	.24**	.29**	.30**
Centrality	.61**	.52**	.56**	.60**
Personal	.42**	.34**	.45**	.46**
Interest	.37**	.39**	.32**	.32**
Obligation	− .18**	− .19**	− .04	− .07
Social	− .02	− .06	− .02	− .01
Doubt	− .04	− .17**	− .25**	− .23**
Tentativeness	− .31**	− .39**	− .36**	− .40**
Dialog	− .05	− .22**	− .23**	− .20**

**p* < 0.05, ***p* < .001.

## Appendix K

Bivariate correlations between religiousness dimensions and centrality of religiousness variables

**Table:**

	Believing	Bonding	Behaving	Belonging
Total	.29**	.41**	.43**	.50**
Interest	.23**	.35**	.38**	.40**
Beliefs	.24**	.29**	.23**	.32**
Prayer	.23**	.26**	.34**	.34**
Experience	.13**	.20**	.19**	.27**
Worship	.28**	.43**	.44**	.52**

**p* < .05, ***p* < .01.

## Appendix L

Bivariate correlations between religiousness dimensions and well-being

**Table:**

	Believing	Bonding	Behaving	Belonging
Well-being				
Total	.06	.13**	.10*	.16**
Emotional	.06	.10*	.09*	.09*
Social	.04	.13**	.10*	.20**
Psychological	.06	.11*	.08	.11*

**p* < .05, ***p* < .01.
